# Efficacy of Biologics on Refractory Eosinophilic Otitis Media Associated with Bronchial Asthma or Severe Uncontrolled CRSwNP

**DOI:** 10.3390/jcm11040926

**Published:** 2022-02-10

**Authors:** Eugenio De Corso, Claudio Montuori, Stefano Settimi, Dario Antonio Mele, Alessandro Cantiani, Marco Corbò, Elena Cantone, Gaetano Paludetti, Jacopo Galli

**Affiliations:** 1 Unit of Otorhinolaryngology—Head and Neck Surgery, “A. Gemelli” Hospital Foundation IRCCS, 00168 Rome, Italy; eugenio.decorso@policlinicogemelli.it (E.D.C.); gaetano.paludetti@unicatt.it (G.P.); jacopo.galli@iol.it (J.G.); 2 Department of Head and Neck and Sensory Organs, Catholic University of the Sacred Hearth, 00168 Rome, Italy; claudio_montuori@libero.it (C.M.); darioantonio.mele@unicatt.it (D.A.M.); cantiani.ale@outlook.it (A.C.); marco.corbo@icloud.com (M.C.); 3 Unit of Otorhinolaryngology, “Federico II” University Hospital, University of Naples “Federico II”, 80131 Naples, Italy; elena.cantone@unina.it

**Keywords:** eosinophilic otitis media, biologics, type-2 inflammation, asthma, chronic rhinosinusitis

## Abstract

Eosinophilic otitis media (EOM) is a difficult-to-treat otitis media characterized by eosinophilic accumulation in the middle ear mucosa and effusion. It is resistant to conventional treatments and strongly associated with asthma and chronic rhinosinusitis with nasal polyps (CRSwNP). The aim of our study is to evaluate the effectiveness of biologics drugs in the control of EOM. This is a retrospective no-profit real-life observational study, involving patients affected by refractory EOM and in treatment with different biologics for concomitant severe eosinophilic asthma or severe uncontrolled CRSwNP (Dupilumab: *n* = 5; Omalizumab: *n* = 1; Mepolizumab: *n* = 1; Benralizumab: *n* = 1). We analyzed data at baseline and at the 6-month follow-up, including specific nasal and otological parameters. We observed an improvement of all nasal outcomes, including NPS, SNOT-22, VAS, and smell function. Regarding specific otological parameters, we observed a significant reduction in the mean value of COMOT-15 score and of Otitis Severity Score at 6-month follow-up compared to baseline (*p* < 0.05). Finally, we observed an improvement in terms of air conduction hearing levels during the treatment. Our results demonstrated that anti type-2 inflammatory pathway biologics can be effective in improving symptoms control and in reducing the severity of eosinophilic otitis media when treating coexisting type-2 diseases, such as asthma and or CRSwNP.

## 1. Introduction

Eosinophilic otitis media (EOM) is a difficult-to-treat otitis media (OM) characterized by eosinophilic accumulation in the middle ear (ME) mucosa and ME effusion with a predominant bilateral prevalence (80%) [1,2]. Eosinophils play a key role in the development of EOM, as well as eosinophilic chronic rhinosinusitis with nasal polyps (eCRSwNP) and severe eosinophilic asthma (SEA) [3,4,5,6]. Therefore, in the clinical practice, it may be observed an association between eosinophilic diseases, such as EOM, eCRSwNP and bronchial asthma [7,8]. Accordingly, previous studies demonstrated that specific anti-eosinophilic treatment may offer benefits on associated linked comorbidities representing different manifestations of a similar disease syndrome [9,10,11].

EOM is not only a refractory and persistent disease, but it also presents a high risk for the development of severe mixed hearing loss or deafness [12]. In fact, the accumulation of highly viscous effusion and granulation with eosinophil infiltration in the middle ear causes bulging of the eardrum and frequently results in its perforation. Therefore, a combination of hearing disturbance with CRSwNP and SEA worsens considerably the quality of life (QoL) [13].

Although EOM in association with ECRS received the attention of the scientific community in the 1990s as new disease entities [13,14], diagnostic criteria were set later in 2011 and implemented with a severity classification [1,2,12]. According to Iino et al. [1], a major criterion (otitis media with effusion or chronic otitis media with eosinophil-dominant effusion) and two or more minor criteria (highly viscous middle ear effusion; resistance to conventional treatment for otitis media; association with bronchial asthma; association with nasal polyposis) are needed to assess the proper diagnosis of EOM. Exclusion criteria are eosinophilic granulomatosis with polyangiitis (EGPA), formerly known as Churg–Strauss syndrome, and hyper eosinophilic syndrome.

EOM is considered a type-2 (Th2) inflammatory disease, as it contains numerous eosinophils and high concentrations of biomarkers of the type-2 inflammation, such as immunoglobulin (Ig)-E, eosinophil cationic protein (ECP), and interleukin (IL)-5 [1,2]. Since current medical treatment, including local and systemic corticosteroids, is notoriously challenging and surgery is often ineffective [13], new therapeutic strategies are required. Biologic drugs, such as anti-IgE and anti-IL-5, anti-IL-4/IL-13 monoclonal antibodies, are currently being used with clinical success in patients with type-2 diseases, such as asthma and CRSwNP [2], and only recently some authors [12] suggested their efficacy also in the treatment of comorbid EOM.

In this study, we report nasal and otological data about patients affected by refractory EOM, treated with different biologics (Omalizumab, Benralizumab, Mepolizumab, and Dupilumab) indicated for concomitant SEA and/or severe uncontrolled CRSwNP. The aim of our study is to evaluate the effectiveness of biologics in the control of difficult-to-treat eosinophilic otitis media optimizing outcomes of treatment.

## 2. Materials and Methods

### 2.1. Study Population and Study Design

This is a retrospective observational no profit study, involving patients affected by refractory EOM and in treatment with biologics for SEA or severe uncontrolled CRSwNP. Cases were selected from medical records of patients in biological treatment and followed at our institution (“A. Gemelli” Hospital Foundation IRCCS, Catholic University of Sacred Heart in Rome) for the comorbid eosinophilic otitis media, between November 2020 and December 2021. Informed consent about privacy and utilization of clinical data was obtained from all patients at the time of the original data collection. Medical data were therefore anonymously analyzed. The study was approved by our institutional review board.

Refractory eosinophilic otitis media was defined according to the diagnostic criteria proposed by Iino et al. [1]. We did not sample middle ear effusion for eosinophil count, as the EOM was clinically defined according to Iino et al.’s criteria in all patients by selecting one out of the two major criteria (otitis media with effusion or chronic otitis media with eosinophil-dominant effusion) plus two or more minor criteria (highly viscous middle ear effusion; resistance to conventional treatment for otitis media; association with bronchial asthma; association with nasal polyposis). SEA was diagnosed according to GINA guidelines [15,16], finally CRSwNP was diagnosed according to European Position Paper on Rhinosinusitis and Nasal Polyps (EPOS 2020) criteria [17,18].

Inclusion criteria were:

- Male or female aged 18–75 years;
- Confirmed diagnosis of refractory eosinophilic otitis media (EOM);
- Ongoing treatment with biologics for SEA and/or severe uncontrolled CRSwNP;
- Willingness and ability to provide written informed consent;
- Observational follow-up period of at least 6 months.

Exclusion criteria were:

- History of genetic, congenital or acquired immunodeficiency;
- Autoimmune diseases;
- Current malignancy;
- Previous radiotherapy for head and neck cancer.

Based on inclusion and exclusion criteria, we included three patients affected by SEA and concomitant CRSwNP, plus EOM in treatment with Mepolizumab (*n* = 1), Omalizumab (*n* = 1) and Benralizumab (*n* = 1). Furthermore, we included 5 patients affected by severe uncontrolled CRSwNP and concomitant EOM and in treatment with Dupilumab. A total number of 8 patients were enrolled in this study.

We analyzed data retrospectively at baseline and at the 6-month follow-up; these data included demographics, SEA and CRSwNP features, biological therapy characteristics, EOM criteria, and therapy administered before biologics and during treatment. At the clinical interviews, we focused on nasal and hearing symptoms, the need for oral corticosteroids (a brief cycle was considered of at least 5 continuous days) and number of surgeries.

Among specific sinonasal clinical features, we took into consideration:

- SNOT-22. We used the validated Italian version of SNOT-22. Possible total score range: 0–110. A SNOT-22 score < 20 was suggestive of mild symptoms. During follow-up time, the minimal clinically important difference (MCID) in SNOT-22 scores was assumed for an 8.9-point increase as reported in previous studies [19].
- Nasal endoscopy (Nasal Polyp Score). Each side of the nasal cavity was separately evaluated and scored in a range from 0–4 (0 = no polyps; 1 = small polyps in the middle meatus not reaching below the inferior border of the middle turbinate; 2 = polyps reaching below the lower border of the middle turbinate; 3 = large polyps reaching the lower border of the inferior turbinate or polyps medial to the middle turbinate; and 4 = large polyps causing complete obstruction of the inferior nasal cavity). The sum of the scores for both nasal cavities was recorded as the NPS value [20].
- VAS for symptoms. Intensity of symptoms was measured on a horizontal 10 cm line. A mean score for each symptom analyzed was obtained using the average value of the scores assigned to all patients for the same symptom [20].
- Cellular infiltration of nasal mucosa at nasal cytology. Nasal leukocyte counts were performed on nasal scraped tissue, obtained from the inferior turbinate bilaterally. Scraping was performed by rhinoprobe (Farmark s.n.c, Milan, Italy). The sample was gently spread on glass slides and immediately fixed in 95% ethyl alcohol and stained with May–Grunwald–Giemsa. The slides were examined under oil immersion by light microscopy at a magnification of 400×. The slides were examined under oil immersion by light microscopy at a magnification of 1000×. Nasal tissue eosinophil infiltration was measured as “eosinophil count per high power field (Ec-hpf)” and reported as the mean of at least 10 high powered fields observed at nasal cytology [21].
- Sniffin’Sticks identification test. It consists of 16 blue pens with black numbers. Each pen is presented only once and an interval of at least 30 s is observed between each presentation to avoid olfactory desensitization. For each odorant pen, the subject must make a forced choice from a list of 4 written proposals. The identification score corresponds to the number of correct responses out of 16 total score. The results were associated with smell function as follows: 0–5 anosmia, 6–11 hyposmia, and 12–16 normal smell [22].

Among specific otologic parameters, we took into consideration:

- Otitis severity score (see Table 1), a scoring system used to evaluate the severity of EOM (Iino et al.). The degree of severity of EOM was evaluated according to five items: (1) quantity of middle ear effusion (MEE) or otorrhea; (2) condition of the middle ear mucosa; (3) frequency of intratympanic injection of triamcinolone acetonide; (4) frequency of administration of systemic corticosteroids; and (5) frequency of administration of antibiotics. These items were scored on a scale from 0 to 2 [23].
- Pure tone average (PTA) at audiometry, average of hearing threshold levels at a set of specified frequencies: typically, 500, 1000, 2000 and 4000 Hz. Patients’ PTA was measured at pure tone audiometry test conducted before biological therapy and after 6 month of follow-up time.
- Chronic otitis media outcome test (COMOT-15). We used the validated Italian version of COMOT-15. The COMOT-15 consists of 3 subscales, categorized as ear symptoms (questions 1–6), hearing function (questions 7–9) and mental health (questions 10–13), which form the overall score. In addition to questions from 1 to 13, the COMOT-15 contains two other questions: a general evaluation of the impact of chronic otitis media on QoL (question 14) and a question on the frequency of ENT visits as a result of chronic otitis media in the previous 6 months (question 15). Possible total score range 0–75 [24].

### 2.2. Statistical Analysis

Statistical analysis was performed using SPSS for Windows (Chicago, IL, USA). Continues values, such as symptoms scores and endoscopic scores, were expressed as mean ± standard deviation (SD). Comparisons between groups were performed by the Mann–Whitney U-test for non-normally distributed data and *t*-test for paired samples in normally distributed values. The results were considered significant for *p*-values < 0.05.

## 3. Results

### 3.1. Baseline Characteristics of the Population

A cohort of eight patients (five females and three males; mean age: 54.38 ± 17.89; range of age 34–64 years) with refractory EOM and concomitant SEA and/or severe CRSwNP in ongoing treatment with biologics was included in this study. Dupilumab was administered as add-on therapy to local corticosteroids in five out of eight patients for severe chronic rhinosinusitis with nasal polyps, uncontrolled by surgery and/or by brief cycles of systemic corticosteroids. In the remaining three patients out of the eight, even affected by CRSwNP, biologics were administered (Omalizumab, Benralizumab and Mepolizumab) mainly for their SEA as add on therapy with inhaled corticosteroids (ICS) with or without long-acting beta 2-adrenergic agonists. 

All the patients concluded the observation period of at least 6 months. Biologics were all well tolerated by all the included patients. Patient characteristics at the time of initiation of biologic therapy and are shown in Table 2. Associated comorbidities, previous need of oral corticosteroids and surgery are reported in Table 3. Finally, according to EOM classification [23], one patient had perforated bilateral eardrums with otorrhea and granulation type EOM, two patients had one side perforated eardrums with granulation type EOM, and finally, the other five had a chronic middle ear effusion. 

### 3.2. Changes in Specific SinoNasal Outcomes, Need of OCS and Antibiotics, and Biomarkers

We reported data about changes in specific sinonasal outcomes in Table 4. All patients suffered from CRSwNP, with a mean NPS score value of 5.38 ± 1.4. After biologic therapy NPS mean value decreased to 1.63 ± 0.98 at the 6 month follow-up and the difference was statistically significant (*p* < 0.05). The frequency of systemic administration of corticosteroids and antibiotics was reduced from three mean brief cycles in the last year to 0 in the 6 months of therapy. None of the patients took OCS simultaneously with the beginning of biological therapy. In addition, they did not have any need for OCS therapy during all biological treatments. A significant difference between local eosinophilia at nasal cytology before and after treatment was observed in all patients and specifically the mean eosinophil count for high power field (Ec-hpf) decrease from a mean value of 30.9 ± 10.2 to 5.5 ± 3.5 (*p* < 0.05). The mean SNOT-22 total score decreased from 69.5 ± 22.38 to 33 ± 22.23 after 6 months of therapy with a significant statistically difference (*p* < 0.05).

A reduction in mean VAS score for specific nasal symptoms was observed and specifically: mean VAS score for obstruction decreased from 7.5 ± 1.3 to 2.3 ± 0.7; for rhinorrhea from 7.1 ± 1.6 to 2 ± 0.7, and for hyposmia from 8 ± 1.6 to 4 ± 2.3. The differences were all statistically significant (*p* < 0.05). Finally, PNIF mean value increased from 61.23 ± 20.31 to 112.5 ± 23.15 (*p* < 0.05). A statistically significant difference with the Sniffin’Sticks identification test pre-biological treatment versus 6 months of follow-up was also found; the mean score of Sniffin’Sticks identification test value increased from 5.75 ± 4.62 before biologics to 11.13 ± 3.04 after 6 months of the follow-up.

### 3.3. Changes in Severity of Otitis during Treatments with Biologics

**Figure 1:**
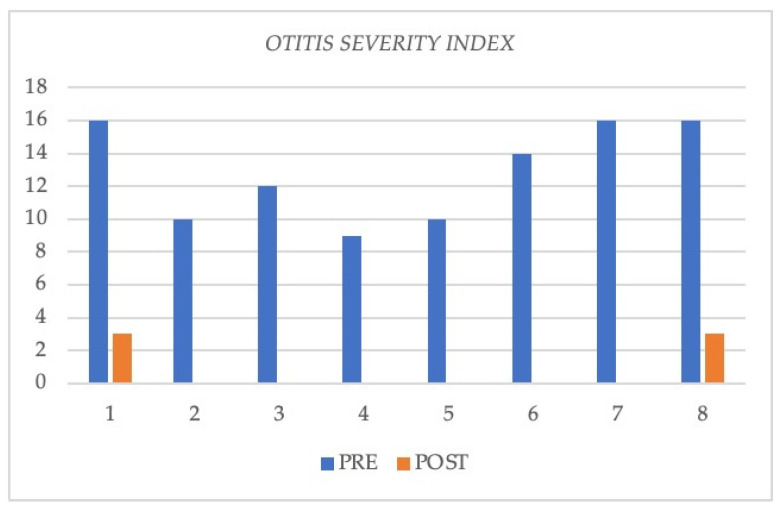
Otitis Severity Index outcomes pre- and post-treatment in all eight patients.

**Figure 2:**
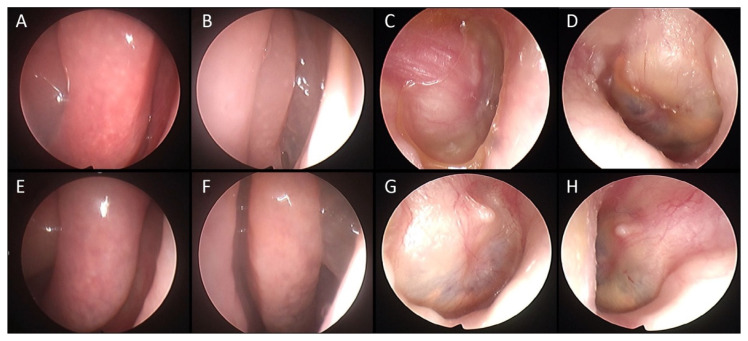
49-year-old patient suffering from SEA and concomitant CRSwNP, with a five-year-old history of EOM unresponsive to conventional treatments, interfering with her social life. She started biological therapy with Mepolizumab, with relief for both asthma and CRSwNP symptoms. At the same time, she experienced a hearing improvement. (**A**) Right nasal cavity with NPS 3 before biologics. (**B**) Left nasal cavity with NPS 3 before biologics. (**C**) Thick edematous eardrum showing effusion type EOM before biological therapy. (**D**) Thick edematous eardrum showing effusion type EOM before biological therapy. (**E**) Right nasal cavity after 6 months of follow-up. (**F**) Left nasal cavity after 6 months of follow-up. (**G**) Right eardrum showing solved otitis after 6 months of follow-up. (**H**) Left eardrum showing solved otitis after 6 months of follow-up.

**Figure 3:**
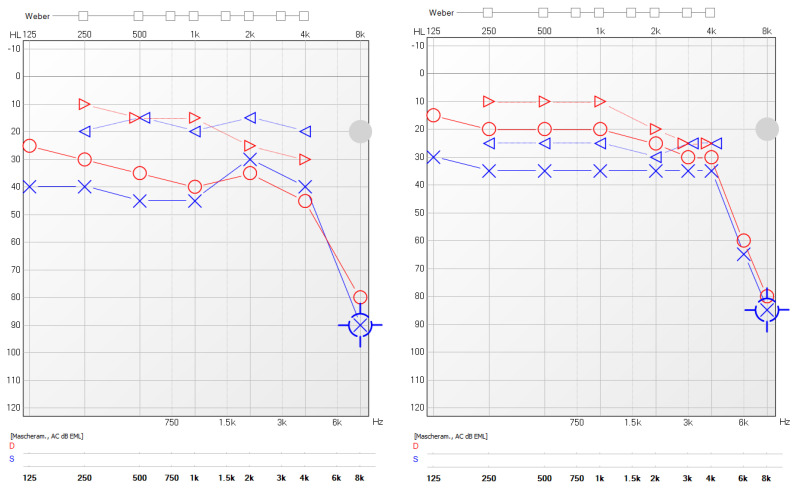
Pure tone audiometry results before biological treatment (Mepolizumab) and after 6 months of follow-up. Red line: right ear; blue line: left ear. ▷: right ear masked bone conduction; ○: right ear air conduction; ◁: left ear masked bone conduction; Ⅹ: left ear air conduction.

**Figure 4:**
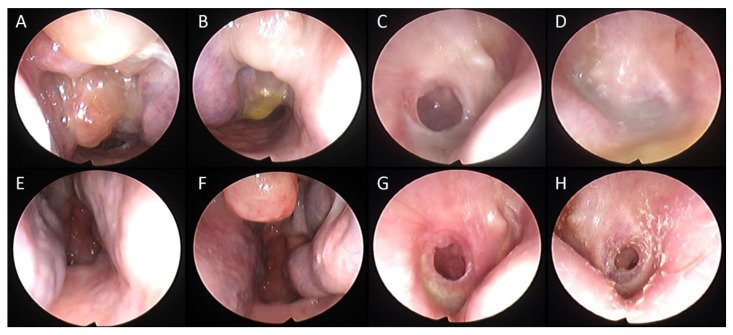
A 54-year-old patient affected by severe uncontrolled CRSwNP and SEA, suffering from intractable EOM with a medical history of progressive bilateral hearing loss, aural fullness, and highly viscous effusion in the right ear, irresponsive to topical and systemic corticosteroid and antibiotic therapy. Biological treatment with Omalizumab had been prescribed by his pulmonologist to treat asthma, with no relief. He was then prescribed with a switch to Dupilumab by our group. Not only did this treatment keep asthma and CRSwNP controlled with a consistent relief of symptoms, but it also led to a remission of EOM. (**A**) Right nasal cavity before biologics. (**B**) Left nasal cavity before biologics. (**C**) Perforated eardrum with otorrhea before biological therapy. (**D**) Perforated eardrum with significant otorrhea before biological therapy. (**E**) Right nasal cavity after 6 months of follow-up. (**F**) Left nasal cavity after 6 months of follow-up. (**G**) Left otoscopic view after six months of treatment. (**H**) Right otoscopic view after six months of treatment.

**Figure 5:**
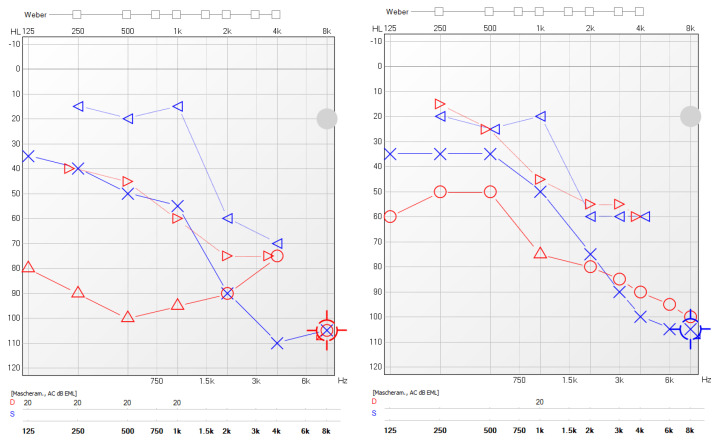
Pure tone audiometry results before biological treatment (Dupilumab) and after 6 months of follow-up. Red line: right ear; blue line: left ear. ▷: right ear masked bone conduction; △: right ear masked air conduction. ○: right ear air conduction; ◁: left ear masked bone conduction; Ⅹ: left ear air conduction.

A significant reduction in the mean value of COMOT-15 score was observed, from 57.63 ± 9.91 to 26 ± 12, and this difference was statistically significant (*p* < 0.05). The values of otitis scores for each patient before and during treatment are reported in Table 5. Furthermore, we observed a significant reduction in Otitis Severity Score, whose values decreased from 12.88 ± 3 at baseline to 0.75 ± 1.39 at 6 months of follow-up time (Figure 1). The difference was statistically significant (*p* < 0.05). Two patients out of eight did not achieve complete resolution in the Otitis Severity Index Score as did the other patients; the first one still had a minimal effusion limited to the mesotympanum, which could be seen through the perforated eardrum, with edematous mucosa in both ears, while the second one had edematous mucosa in both ears and middle ear effusion with intratympanic aeration without eardrum perforation. In Figure 2 and Figure 3, we highlighted the improvement in terms of endoscopic and otoscopic features and in terms of hearing function in the one patient treated with Mepolizumab. In Figure 4 and Figure 5, we highlighted the improvement in terms of endoscopic and otoscopic features and in terms of hearing function in one of the patients treated with Dupilumab.

### 3.4. Efficacy of Biologics on Hearing Function

We observed a decrease in mean air conduction hearing levels at the time of the beginning of biologics and after treatment as shown in Table 5. PTA mean values before therapy were 59.1 ± 14.63 dB for the right ears and 42.25 ± 17.27 dB for the left ears and they decreased to 39.69 ± 16.56 dB for the right ears and 33.99 ± 13.47 dB for the left ears at 6 months follow-up. The difference was not statistically significant (*p* > 0.05). PTA mean value for ears with effusion type EOM went from 55.74 ± 9.25 dB to 33.21 ± 10.44 dB. PTA mean value for ears with granulation type EOM decreased from 68.75 ± 17.59 dB to 54.25 ± 18.02 dB. The difference was not statistically significant (*p* > 0.05) in both cases. No deterioration in bone conduction hearing levels happened in any patient during biologics therapy.

## 4. Discussion

The link between upper and lower respiratory tract physiopathology has been widely described in the literature, and previous clinical and experimental investigations suggested the hypothesis of the unity of upper and lower airways (united airways disease), including middle ear mucosa [25]. This relationship has been confirmed by epidemiological observations, functional and immunological evidence and, indirectly, by noting the effects of drugs used to treat upper respiratory diseases on lower respiratory diseases and vice-versa [25]. In addition, eosinophilic inflammation plays a crucial role in the development of EOM and eCRS, which are reported to be associated [25], usually coexisting with severe asthma [12,26].

Since multiple cytokines, including IL-4, IL-13, and IL-5, are involved in the eosinophilic type-2 inflammation, various molecular targeted drugs have been used in the treatment of patients with allergic or eosinophilic diseases and were introduced as a treatment modality for type-2 refractory disorders [12,13,27]. In this scenario, the success in targeting specific immunologic mediators in asthma with biological drugs has led to an interest in the use of a similar therapeutic approach as an adjunct treatment for CRSwNP [28]. So, monoclonal antibodies, such as anti-IgE (Omalizumab), anti-IL-5 (Mepolizumab, Benralizumab, Reslizumab), and anti-IL-4 and IL-13 (Dupilumab), have emerged as effective treatments for type-2-inflammation-related diseases [26], including EOM, a condition often resistant to conventional treatments (leukotriene receptor antagonist (LTRA), mucoregulator, macrolide antibiotics, topical application of corticosteroids, thus requiring oral corticosteroids (OCS)), with frequent recurrence in the case of treatment suspension [13]. Furthermore, previous works suggest that the improvement of EOM could be reached by optimizing asthma treatment, including corticosteroids; nevertheless, it is well known that long term use of the last one may lead to higher risk of serious adverse effects [29].

Biological drugs, as a new strategy to treat EOM, represent a developing field. However, the related literature consists only of case reports and a few small case series. Even though the use of biologics is not approved for EOM yet, these recent reports have shown that they may be effective against EOM associated with other different type-2 diseases, especially in refractory ones [30]. Previous studies firstly focused on anti-IgE and anti-IL-5 therapies to treat EOM in association with asthma and/or CRSwNP. Authors [31] demonstrated that after anti-IgE treatment, not only asthma, but also hearing loss improved and bone conduction hearing was stable for an extended period with anti-IgE monoclonal antibodies. Literature data demonstrated that anti-IL-5 monoclonal antibodies may improve not only symptoms, but also the QoL of severe asthma, ECRS, and EOM [32,33]. Nevertheless, efficacy of treatments was quite limited in patients with the most refractory and resistant form of EOM, and especially in the granulation type, in which the mechanism responsible for highly granulated mucosa has not been determined yet [12]. Hamada et al. hypothesized that one biologic alone cannot control both asthma and comorbidities, such as eCRS and EOM, suggesting that therapy with two biologics may create “multi-super-responders” in the management and treatment of severe asthma, together with eCRS and EOM [26]. Finally, it has been proposed that the early initiation of therapy might inhibit the progression of EOM [34]. 

A recent study of Iino et al. [12] reported that also long-term add-on anti-IL-4 and IL-13 therapy may improve the clinical condition of EOM in patients unresponsive to the other treatments, including other molecular targeted therapy. Similarly, van der Lans [2] showed that anti-IL-4/IL-13 treatment resulted in a complete and enduring remission of EOM, enabling adequate hearing rehabilitation; concurrent control of the comorbid asthma and CRSwNP was obtained. 

In our study, we described the efficacy of different biologics on refractory eosinophilic otitis media associated with SEA and/or severe uncontrolled CRSwNP. To the best of our knowledge in our sample, although small, we reported the highest number of patients treated with anti-IL-4 and IL-13 in the literature. We observed an improvement, during therapy with biologics, in all considered endpoints. In particular, our data showed a statistically significant reduction in the NPS value after treatment, confirming the ability of biologics to reduce the size of polyps. This improvement was not only in polyp volume, but also in nasal function and inflammation, as demonstrated by the higher values of PNIF and the reduction in local eosinophilia at nasal cytology. A great improvement in terms of quality of life was observed with a significant reduction in the SNOT-22 total score. Finally, the frequency of systemic administration of corticosteroids and antibiotics was reduced from three mean brief cycles in the last year to 0 in the 6 months of therapy. 

The most interesting and original finding concerns the severity of otitis and hearing function. In fact, we observed a significant reduction in the mean score both in the Otitis Severity Score and in the COMOT-15; regarding the latter, all three subscales (ear symptoms, hearing function and mental health) reported an improvement in the subjective symptoms. A concomitant decrease in mean air conduction hearing levels was found, even though the differences between the pre-treatment and post-treatment PTA were not statistically significant; this could be due to the limited number of patients enrolled. Furthermore, a longer administration of biologics over six months may offer some advantages in terms of hearing function. Finally, a direct comparative analysis between different biologics was not possible due to the limited number of patients, but a future study may demonstrate that different biologics may be related to different outcomes in terms of the improvement of hearing function. 

In addition to the small sample size, our study has other limitations that should be mentioned. Firstly, our series included a small sample size, patients were treated with different biologics and were not administered with the same standard of care. Secondly, we did not make any assumptions on ear imaging because it was not part of our diagnostic flow-chart when all patients came to our attention for the first time, since they started biological therapy for asthma and/or CRSwNP, and therefore eosinophilic otitis media was an associated comorbidity. Finally, the 6 month follow-up is not sufficient to evaluate the long-term efficacy of the therapy for the control of EOM. Further studies with larger cohorts of patients, including different subtypes of EOM and longer follow-ups, should be performed to confirm our preliminary suggestions.

## 5. Conclusions

Our results demonstrated that biologics against type-2 inflammation can be effective in improving outcomes in EOM therapy offering a good control of otologic symptoms when treating coexisting type-2 diseases, such as asthma and or CRSwNP. Future observational studies on larger series and randomized control trials should confirm our preliminary results. In this scenario, difficult-to-treat EOM patients could represent a new indication and/or a supplementary diagnostic criterion for treatment with anti-type-2 biologics. 

## Figures and Tables

**Table 1:** Severity scores of eosinophilic otitis media.

Mucosal Condition	
0	Nearly normal or slightly edematous
1	Edematous or slightly thickened
2	Highly thickened or granulated to an extent beyond the position of a normal eardrum
**Quantity of MEE/otorrhea**	
0	No MEE ^1^
1	MEE with intratympanic aeration in a case without eardrum Perforation or otorrhea limited to the mesotympanum in a case with perforation
2	Mesotympanum totally filled with MEE in a case without perforation or otorrhea coming out from the mesotympanum to the external auditory canal in a case with perforation
**Frequency of intratympanic administration of corticosteroid**	
0	None
1	Once in the previous 3 months
2	Two or more times in the previous 3 months
**Frequency of systemic administration of corticosteroids**	
0	None.
1	7 days or less in the previous 3 months.
2	More than 7 days in the previous 3 months.
**Frequency of systemic antibiotics**	
0	None.
1	7 days or less in the previous 3 months.
2	More than 7 days in the previous 3 months.

^1^ Abbreviations. MEE: middle ear effusion.

**Table 2:** Cases series: epidemiology and phenotyping.

Epidemiology	
Age	54.38 ± 17.89; range 34–64 years
Female	5/8 (62.5%)
Male	3/8 (37.5%)
**Phenotyping**	
Concomitant allergy	6/8 (75%)
Concomitant CRSwNP	8/8 (100%)
Concomitant asthma Peripheral blood hyper-eosinophilia Previous sinonasal surgery	8/8 (100%) 6/8 (75%) 8/8 (100%)
ASA sensitivity Smoking SNOT-22, mean Nasal polyp score, mean	1/8 (12.5%) 0/8 69.5 ± 22.39 5.38 ± 1.4
Sniffin’Sticks identification test, mean PNIF, mean Otorrhea Perforated ear drum Middle ear effusion PTA at baseline mean right PTA at baseline mean left COMOT-15, mean Otitis severity score, mean	5.75 ± 4.62 61.23 ± 20.31 3/8 (37.5%) 3/8 (37.5%) 5/8 (62.5%) 59.1 ± 14.63 42.25 ± 17.27 57.63 ± 9.91 12.88 ± 3

Abbreviations. CRSwNP: chronic rhinosinusitis with nasal polyps; ASA: aminosalicylic acid; SNOT-22: sinonasal outcome test; PNIF: peak of nasal inspiratory flow; PTA: pure tone average; COMOT-15: chronic otitis media outcome test.

**Table 3:** Clinical characteristics of patients.

Case	Sex, Age	Ongoing Biologics	Primary Indication for Biologics	Previous Treatments in the Last Years	Previous Surgeries	Associated Diseases	Type of EOM
1	M, 64	Dupilumab	Severe uncontrolled CRSwNP	3 brief cycles of OCS and antibiotics, previous treatment with omalizumab	Almost 20 NS	Mild–moderate asthma	Bilateral perforation
2	F, 63	Dupilumab	Severe uncontrolled CRSwNP	4 brief cycles of OCS, antibiotics	1 NS	Mild moderate asthma	COM
3	F, 49	Mepolizumab	SEA	3 brief cycles of OCS, antibiotics	2 NS	CRSwNP Severe hyper eosinophilia	COM
4	M, 57	Benralizumab	SEA	2 brief cycles of OCS, antibiotics	2 NS	CRSwNP	COM
5	M, 62	Omalizumab	SEA	2 brief cycles of OCS, antibiotics	7 NS	CRSwNP	COM
6	F, 48	Dupilumab	Severe uncontrolled CRSwNP	2 brief cycles of OCS, antibiotics	2 NS	Aspirin-intolerant Mild–moderate asthma	Monolateral perforation with granulation
7	F, 34	Dupilumab	Severe uncontrolled CRSwNP	More than 3 brief cycles of OCS, antibiotics	1 NS >2 ES	Mild–moderate asthma	Monolateral perforation with granulation
8	F, 58	Dupilumab	Severe uncontrolled CRSwNP	More than 3 brief cycles of OCS, antibiotics	1 NS	Mild–moderate asthma	COM

Abbreviations. M: male; F: female; OCS: oral corticosteroids; EOM: eosinophilic otitis media; COM: chronic otitis media; NS: nasal surgery; ES: ear surgery (bilateral tympanostomy with T-tube insertion and 2-stage tympanoplasty in right ear).

**Table 4:** Specific sinonasal outcomes pre- and post-treatment with biologics.

Case	Sex, Age	NPS Pre	NPS Post	SNOT-22 Pre	SNOT-22 Post	Sniffin’Sticks Pre-IT	Sniffin’Sticks Post-IT	PNIF Pre (L/min)	PNIF Post (L/min)
1	M, 64	8	4	63	15	0	6	40	150
2	F, 63	6	0	81	35	0	8	50	100
3	F, 49	4	1	88	54	11	14	50	100
4	M, 57	4	0	80	16	4	13	50	100
5	M, 62	4	1	81	36	4	9	50	100
6	F, 48	6	2	21	10	6	12	80	100
7	F, 34	5	2	57	23	12	14	70	100
8	F, 58	6	3	85	75	9	13	100	150

Abbreviations. NPS: nasal polyp score; SNOT-22: sinonasal outcome test; PNIF: peak nasal inspiratory flow; IT: identification test.

**Table 5:** Specific sinonasal outcomes pre- and post-treatment with biologics.

Case	Sex, Age	Otitis Severity Index Pre	Otitis Severity Index Post	PTA Pre	PTA Post	Cycles of OCS/Antibiotics Need Pre	Cycles of OCS/Antibiotics Need Post	COMOT 15 Pre	COMOT 15 Post
1	M, 64	16	3	90 R 76.25 L	73.75 R 65 L	3 brief cycles of OCS and antibiotics, previous treatment with omalizumab	No	63	34
2	F, 63	10	0	65.25 R 60.5 L	35.3 R 33.2 L	4 brief cycles of OCS, antibiotics	No	56	15
3	F, 49	12	0	40 R 38.75 L	23.75 R 35 L	3 brief cycles of OCS, antibiotics	No	65	29
4	M, 57	9	0	51.2 R 37.5 L	29.2 R 32.5 L	2 brief cycles of OCS, antibiotics	No	46	5
5	M, 62	10	0	55 R 30 L	25.3 R 20 L	2 brief cycles of OCS, antibiotics	No	47	19
6	F, 48	14	0	56.25 R 32.5 L	40.5 R 28.75 L	2 brief cycles of OCS, antibiotics	No	47	24
7	F, 34	16	0	52.5 R 37.5 L	37.25 R 32.5 L	>3 brief cycles of OCS, antibiotics	No	67	43
8	F, 58	16	3	62.5 R 25 L	52.5 R 25 L	>3 brief cycles of OCS, antibiotics	No	70	39

R: right, L: left; PTA: pure tone average; OCS: oral corticosteroids, COMOT-15: chronic otitis media outcome test.

## Data Availability

The data presented in this study are available on reasonably request from the corresponding author.
